# Efficacy of ivermectin for malaria vector control: a systematic review and meta-analysis of randomized clinical trials

**DOI:** 10.1186/s12936-026-05895-z

**Published:** 2026-03-31

**Authors:** Shrouk Ramadan, Ahmed Talkhan, Anas Mansour, Dua’a Kanaan, Ahmed Noureldeen Abbas, Abdelrahman Awad, Omer Bin Sahel, Lara Hamzeh Hamzeh, Ayat Alfaki, Mohamed Hany, Mohamed Elnouty, Mohammed Hammad Jaber Amin

**Affiliations:** 1 Faculty of Medicine, Ain Shams University, Cairo, Egypt; 2 Medical Research Group of Egypt (MRGE), Negida Academy, Arlington, MA USA; 3 Faculty of Medicine, Mansoura University, Mansoura, Egypt; 4 Faculty of Medicine, Al-Azhar University, Cairo, Egypt; 5 MBBS,School of Medicine, The University of Jordan, Amman, Jordan; 6 Faculty of Medicine, Minia University, Minia, Egypt; 7 University of Padova, Padua, Italy; 8 Faculty of Medicine, Seiyun University, Seiyun, Yemen; 9 Faculty of Medicine, Caucasus International University, Tbilisi, Georgia; 10 Faculty of Medicine and Health Science, Omdurman Islamic University, Omdurman, Sudan; 11 School of Medicine, Newgiza University, Giza, Egypt; 12 Mayo Clinic, Scottsdale, AZ USA; 13 Department of Medicine, Alzaiem Alazhari University, 123 Alkalakla, Khartoum, Sudan

**Keywords:** Malaria, Ivermectin, Systematic review, Meta-analysis, Randomized controlled trials, Mosquito mortality, Vector control

## Abstract

**Background:**

Despite global malaria control efforts, malaria continues to cause approximately 241–249 million cases and over 600,000 deaths annually. Ivermectin, a broad-spectrum antiparasitic drug with endectocidal activity against mosquitoes feeding on treated hosts, has been proposed as a potential malaria vector-control intervention.

**Objective:**

To systematically evaluate the effectiveness of ivermectin in reducing malaria transmission outcomes in adult populations, including malaria incidence, prevalence, mosquito mortality, and safety outcomes.

**Methods:**

Following PRISMA 2020 guidelines, we included randomized controlled trials (RCTs) and cluster-RCTs evaluating ivermectin compared with placebo, standard care, or non-ivermectin controls in adult populations. Outcomes included malaria incidence, prevalence, mosquito mortality, and adverse events.

**Results:**

Ten randomized trials involving 63,192 participants were included. Community-level analyses showed no significant reduction in malaria prevalence, with very wide confidence intervals reflecting substantial imprecision. Entomological trials demonstrated increased mosquito mortality following ivermectin exposure (RR 1.89; 95% CI 1.26–2.83), although heterogeneity was substantial (I^2^ > 97%). Across studies, ivermectin was generally well tolerated, with no significant increase in adverse events.

**Conclusions:**

Current evidence does not demonstrate consistent reductions in malaria incidence or prevalence with ivermectin administration, although laboratory and experimental studies suggest mosquitocidal effects. Further large, well-designed cluster-randomized trials are required to determine whether ivermectin can contribute to malaria control strategies.

**Supplementary Information:**

The online version contains supplementary material available at 10.1186/s12936-026-05895-z.

## Key points

● Ivermectin has been proposed as a novel malaria vector-control strategy due to its endectocidal effects on mosquitoes.

● In this review, we included ten randomized trials that looked at how ivermectin affects malaria outcomes in different settings.

● Overall, meta-analysis showed no significant effect on malaria prevalence/incidence; entomological trials showed significant mosquito mortality (RR 1.89).

## Introduction

Malaria is a life-threatening disease caused by *Plasmodium* parasites transmitted to humans by infected female *Anopheles* mosquitoes [1, 2]. Despite major global control efforts, it remains one of the most destructive vector-borne diseases, accounting for approximately 241–249 million cases and over 600,000 deaths annually [3].

The control of malaria relies on a multifaceted strategy targeting both the mosquito vector and the *Plasmodium* parasite. Current interventions include vector management through long-lasting insecticidal nets (LLINs) and indoor residual spraying (IRS), antimalarial treatment and prophylaxis, vaccination, and strengthened surveillance with rapid diagnostic tools [4, 5]. These measures have substantially reduced malaria incidence and mortality in many endemic regions.

However, the sustainability of these gains is increasingly threatened [6, 7]. The widespread resistance of *Anopheles* mosquitoes to pyrethroids and other insecticides has significantly weakened the effectiveness of LLINs and IRS, while growing resistance of *Plasmodium* species to artemisinin-based combination therapies (ACTs) poses a major challenge to treatment success and threatens to reverse recent progress [8, 9].

In response to these challenges, attention has shifted toward novel or complementary interventions that act through distinct mechanisms. Ivermectin, a broad-spectrum antiparasitic first developed for animals and later used to treat human infections like onchocerciasis and lymphatic filariasis, has drawn strong interest for malaria vector control. Its ability to kill both internal and external parasites makes it a promising tool for new malaria control strategies and other emerging therapies [10, 11].

Some recent trials have investigated ivermectin’s potential in malaria control, but their findings have been inconsistent. For example, a study conducted in Kwale and Kenya [12] reported a 26% reduction in malaria incidence with ivermectin versus albendazole and no safety concerns. Another study in southwest Burkina Faso [13] found that repeated high-dose ivermectin MDA with seasonal malaria chemoprevention did not significantly reduce incidence but decreased mosquito survivorship and improved hemoglobin levels.

Despite these encouraging but mixed findings, the overall evidence regarding ivermectin’s effect on malaria transmission remains inconclusive. Previous evidence, including a Cochrane systematic review [14], suggested potential mosquitocidal and transmission-reducing properties of ivermectin; however, the conclusions were limited by the inclusion of only one eligible cluster-randomized controlled trial in the quantitative synthesis and substantial methodological constraints. In addition, the Cochrane review restricted inclusion to cluster-randomized trials, excluding individual-level randomized studies. Since its publication, several new randomized trials across diverse endemic settings have become available. These trials expand the evidence base and provide an opportunity for a more comprehensive and updated assessment of ivermectin’s role in malaria control. Since the publication of these earlier reviews, several new randomized clinical trials have been conducted across diverse malaria-endemic settings. These recent trials significantly expand the evidence base and address several of the earlier limitations, thereby creating a timely need for an updated and comprehensive meta-analysis. Incorporating these new data is essential to a more accurate determination of ivermectin’s potential role in malaria control and to guide future policy and implementation strategies.

## Methods

### Protocol registration, data source and search strategy

This systematic review was designed, conducted, and reported in accordance with the Cochrane Handbook for Systematic Reviews of Interventions (version 6.4) and PRISMA 2020 guidelines [15, 16]. The PROSPERO protocol was updated during the conduct of the review to Key deviations included the addition of mosquito mortality as an outcome, inclusion of individual-level RCTs, and incorporation of quantitative synthesis methods not specified in the original protocol. These deviations are transparently reported in the manuscript and supplementary materials. *ID:CRD420261341512.*

We systematically searched PubMed, Scopus, Web of Science, and the Cochrane Library from their inception through August 2025. Detailed search terms and strategies are provided in Supplementary Table 1. Duplicate records were removed prior to screening (Table 1).

**Table 1:** Baseline characteristics of the patients included in the studies

Study (Year)	Treatment group	Number of Patients	Age, Mean(SD)	Male, N(%)	BMI, Mean(SD)	Height (cm), Mean(SD)	Weight (kg/m^2^), Mean(SD)	Hb (g/dL), Mean(SD)	Vivax (%)	Ovale (%)	Falc. (%)	Parasitaemia, parasites/μl, Mean(SD)
Mbassi 2023 [26]	IVM 0.2 mg/kg × 1d	5	27.67 (19.11)	3 (60)	19.2 (0.5)	167.33 (7.04)	56.67 (9.05)	—	0	0	41	308 (75)
	IVM 0.2 mg/kg × 2d	5	25.33 (18.1)	3 (60)	23.07 (8.85)	163.67 (12.07)	62.47 (18.1)	—	0	0	41	560 (552)
	IVM 0.2 mg/kg × 3d	5	39.33 (32.18)	2 (40)	26.73 (2.92)	158.33 (15.08)	64.67 (12.07)	—	0	0	41	405 (303)
	IVM 0.3 mg/kg	17	26.33 (9.7)	10 (59)	22.47 (2.51)	160.67 (12.13)	62.67 (9.7)	—	0	0	41	457 (331)
	placebo	17	27.67 (16.98)	13 (76)	22.83 (3.8)	168 (8.08)	61.67 (5.66)	—	0	0	41	1,378 (1,450)
Smit 2019 [25]	IVM 600 µg/kg/d	22	27.3 (7.4)	13 (59)	22.9 (3.4)	—	—	—	—	—	100	—
	IVM 300 µg/kg/d	24	25.5 (7.5)	16 (67)	21.5 (3.0)	—	—	—	—	—	100	—
	placebo	23	26.0 (5.0)	14 (61)	21.6 (2.6)	—	—	—	—	—	100	—
Smit 2018 [24]	IVM 600 µg/kg/d	47	25.4 (6)	27 (57)	22.5 (2.9)	—	—	14.1 (2.2)	—	—	—	—
	IVM 300 µg/kg/d	48	25.5 (6.9)	29 (60)	22 (3.1)	—	—	14.2 (1.8)	—	—	—	—
	placebo	46	25.1 (5)	27 (59)	22.3 (2.8)	—	—	13.9 (1.7)	—	—	—	—
Metzger 2020 [23]	IVM 0.4 mg/kg	8	27 (4.91)	4 (50)	23 (3.51)	171 (6.31)	69 (14.0)	—	—	—	100	—
	placebo	4	30 (4.37)	2 (50)	24 (4.37)	167 (10.2)	68 (21.6)	—	—	—	100	—
Ouédraogo 2015 [22]	AL-IVM1	40	17 (3.84)	18 (45)	—	—	—	13.13 (1.23)	0	0	0.5	106 (135)
	AL-IVM2	40	17.17 (1.92)	28 (70)	—	—	—	13 (1.15)	0	0	1.2	195 (278)
	AL alone	40	18.6 (4.08)	26 (65)	—	—	—	13.23 (1.85)	0	0	0.7	123 (141)
Chaccour 2025 [21]	IVM 0.4 mg/kg	15,195	24.5 (21.54)	—	—	—	—	—	—	—	—	—
	Albendazole	7,529	24.2 (13.48)	—	—	—	—	—	—	—	—	—
Some 2025 [13]	Ivermectin	1,928	16 (4.47)	966 (50.1)	—	—	—	—	—	—	—	—
	Placebo	1,604	16 (4.47)	759 (47.3)	—	—	—	—	—	—	—	—
Hutchins 2025 [20]	IVM 300 µg/kg × 3d	13,832	20 (20.7)	6,836 (49.4)	—	—	—	—	0	1	99	—
	placebo	12,050	20 (20.7)	5,951 (49.4)	—	—	—	—	0	1	99	—
Dabira 2022 [19]	Intervention	4,939	16.67 (18.54) †	620 (42)	—	—	—	—	0	0	100	—
	control	5,699	16.33 (17.8) †	518 (43)	—	—	—	—	0	0	100	—
Chaccour 2010 [18]	Ivermectin	13	—	—	—	—	—	—	—	—	—	—
	Control	12	—	—	—	—	—	—	—	—	—	—

IVM: ivermectin; AL-IVM: artemether-lumefantrine + ivermectin; Falc: Plasmodium falciparum

Continuous variables are reported as mean (SD); categorical variables are N (%) unless stated otherwise

— indicates the study did not report this outcome

Parasitemia values are in parasites/µL, reported as mean (SD)

Age strata were used in some studies (< 5, 5–14, > 15 y); mean (SD) calculated across all ages

Species distribution columns indicate the percentage of malaria-positive participants infected with each Plasmodium species

Total sample sizes are reported per arm; for cluster trials, values reflect individual participantsIVM: ivermectin; AL-IVM: artemether-lumefantrine + ivermectin; Falc: Plasmodium falciparum

Continuous variables are reported as mean (SD); categorical variables are N (%) unless stated otherwise

— indicates the study did not report this outcome

Parasitemia values are in parasites/µL, reported as mean (SD)

Age strata were used in some studies (< 5, 5–14, > 15 y); mean (SD) calculated across all ages

Species distribution columns indicate the percentage of malaria-positive participants infected with each Plasmodium species

Total sample sizes are reported per arm; for cluster trials, values reflect individual participantsIVM: ivermectin; AL-IVM: artemether-lumefantrine + ivermectin; Falc: Plasmodium falciparum

Continuous variables are reported as mean (SD); categorical variables are N (%) unless stated otherwise

— indicates the study did not report this outcome

Parasitemia values are in parasites/µL, reported as mean (SD)

Age strata were used in some studies (< 5, 5–14, > 15 y); mean (SD) calculated across all ages

Species distribution columns indicate the percentage of malaria-positive participants infected with each Plasmodium species

Total sample sizes are reported per arm; for cluster trials, values reflect individual participants

### Eligibility criteria

Studies met stratified PICOS criteria according to trial type. For individual-level clinical trials, the population included malaria-infected participants with confirmed parasitemia. The intervention was ivermectin monotherapy or combination therapy (150–600 µg/kg), compared against placebo, standard antimalarial therapy, or non-ivermectin controls. Primary outcomes included parasitemia incidence over 21–28 days or time-to-parasitemia, and the design was restricted to randomized controlled trials (RCTs). A full list of excluded studies with reasons is provided in Supplementary Table 2**.**

For community-level MDA or cluster-randomized trials, eligible populations consisted of all-age community residents irrespective of infection status. Interventions included mass drug administration or cluster-randomized ivermectin programs compared with placebo, standard care, or no ivermectin. Primary outcomes were community-level malaria incidence over 6–24 months or prevalence measured at defined endpoints.

For entomological trials, eligible studies assessed *Anopheles* mosquito mortality or survival after feeding on ivermectin-treated hosts. The intervention consisted of ivermectin-treated blood meals compared with untreated control meals, with outcomes measured as mosquito mortality within 3–7 days post-feeding using cone or direct-feeding assays.

Eligible designs were limited to RCTs and cluster-RCTs. Exclusions comprised non-English publications, reviews, observational designs, conference abstracts, and in vitro studies.

### Trial categorization and synthesis strategy

Included trials were stratified into three non-overlapping categories. Category 1 (individual-level clinical trials, n = 3) comprised short-term studies (< 1 month) with individual randomization among malaria-infected participants. Category 2 (community-level MDA/cluster trials, n = 4) involved population-based interventions with long-term follow-up (> 6 months) and cluster randomization. Category 3 (entomological trials, n = 3) consisted solely of mosquito mortality assays.

Studies reporting outcomes only as model-derived incidence estimates without sufficient data to calculate comparable effect measures were summarized narratively and not included in quantitative synthesis.

No meta-analyses crossed categories due to differences in denominator units (individual versus community), follow-up duration, outcome structure, or analytic adjustment requirements. Separate random-effects meta-analyses were conducted within comparable trial types, while narrative synthesis was applied for heterogeneous outcomes.

### Study selection

**Fig. 1:**
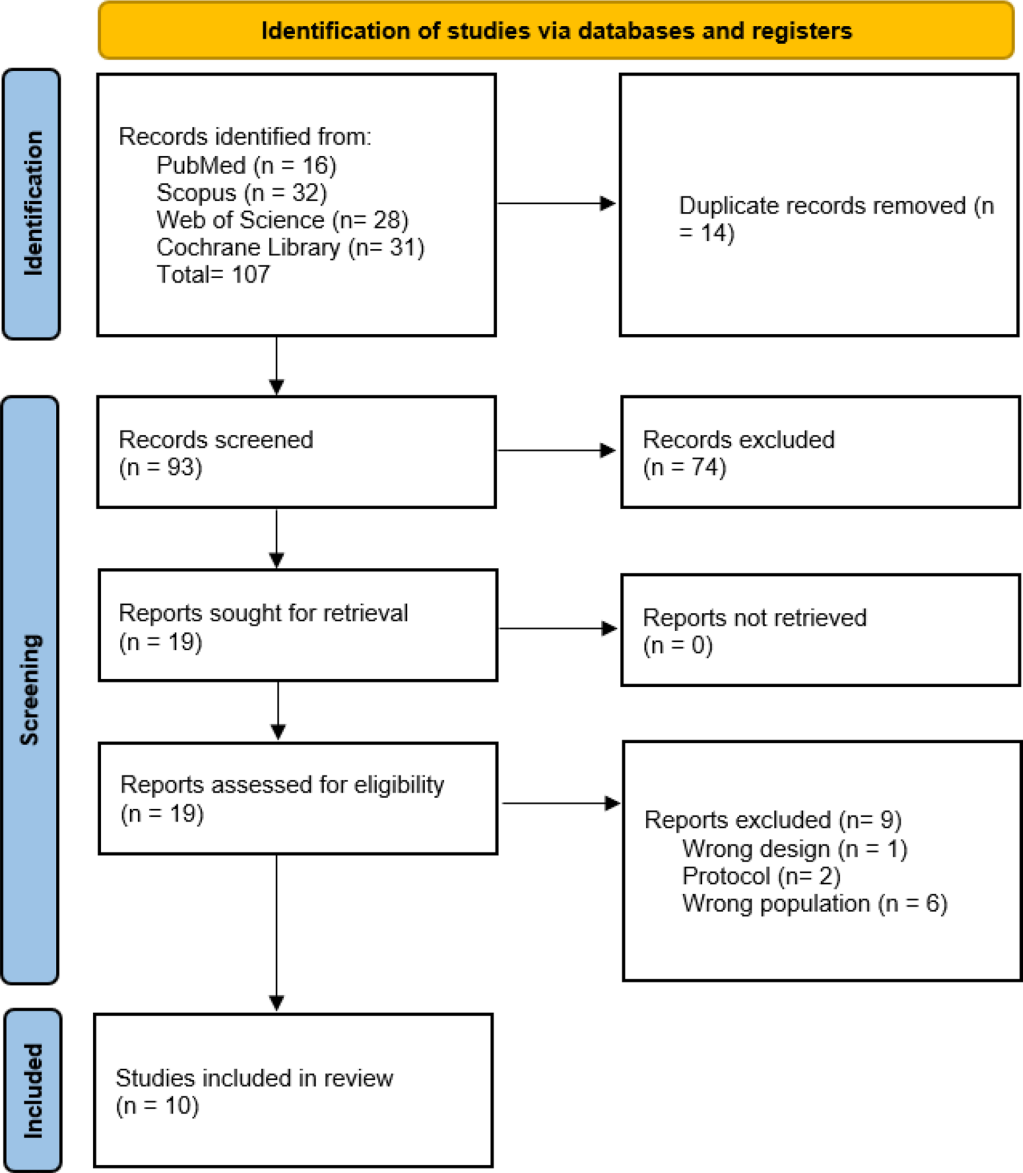
PRISMA 2020 flow diagram for new systematic reviews which included searches of databases

All retrieved records underwent deduplication in EndNote and were then exported to Rayyan for screening. Four reviewers independently screened titles and abstracts in pairs using predefined inclusion and exclusion criteria Each full-text article was assessed independently by two reviewers. Full-text screening was conducted independently by the same reviewers, with disagreements resolved through discussion or adjudication by a third reviewer. The study selection process is summarized in the PRISMA 2020 flow diagram **(**Fig. 1**).**

### Data extraction

Two reviewers independently extracted key trial characteristics, including category, design, geographic site, study period, follow-up duration, sample size per arm, ivermectin dose, regimen, route, and co-administered medications. Baseline participant characteristics (age, sex, hemoglobin, parasitemia levels) were also extracted, along with category-specific outcomes—namely incidence (events/total), prevalence (events/total), and mosquito mortality percentages. Data extraction followed a categorized framework (Table 2). Discrepancies were resolved via consensus.

**Table 2:** Summary of Included Studies

Study ID	Conduct Year Not Published	Study design	Trial Type infected-person trials, community MDA trials, and entomological trials,	NCT/Protocol	Site	Follow-up (Weeks)	Total N	Arms (Dose, n)	Population	Primary Endpoint in short words from abstract	Efficacy & Adverse Events in short words from abstract
Mbassi [26]	2019–2020	Multiple-dose stage, randomized, double-blind, placebo-controlled phase Ib/IIa, monocentric	Infected-person, clinical	PACTR201908520097051	Gabon	1	49	0.2 mg/kg × 1 day (n = 5); 0.2 mg/kg × 2 days (n = 5); 0.2 mg/kg × 3 days (n = 5); 0.3 mg/kg × 3 days (n = 17); Placebo (n = 17)	Adults 18 yrs, P. falciparum 200–5000/µL, afebrile	The time required to achieve and sustain a marked reduction in parasitaemia was assessed	All regimens produced marked parasitaemia reduction without a dose-dependent effect, one showed faster onset, and adverse events were frequent but mild to moderate with no serious events
Smit [24]	2015–2016	RCT, double-blind, 3 arms	Clinical/Entomology	NCT02511353	Kisumu, Kenya	4	141	600 µg/kg/day × 3 (n = 46); 300 µg/kg/day × 3 (n = 48); Placebo (n = 47)	Adults with uncomplicated malaria + DP	Mosquito mortality following feeding was assessed using both membrane-based and skin-based methods	No difference membrane vs skin feeding. No SAEs. Mild AEs: headache, dizziness, GI upset
Smit [25]	2019–2020	Randomised, double-blind, placebo-controlled, parallel 3-arm, phase II	Clinical/Entomology	NCT02511353	Kisumu, Kenya	4	141	600 µg/kg/day × 3 (n = 47); 300 µg/kg/day × 3 (n = 48); Placebo (n = 46)	Adults 18–50 yrs, uncomplicated P. falciparum + DP	A persistent mosquitocidal effect was observed, indicating sustained activity over time	Significant increase in mosquito mortality vs placebo. AEs more frequent: pupil changes, Hb, ALT/AST, GI, nervous, vascular
Metzegar [23]	2018	Randomized controlled human malaria infection trial	Infected-person, clinical	EudraCT2017-002723–16	Tübingen, Germany	Day 35 & 90	12	0.4 mg/kg single (n = 8); Placebo (n = 4)	Healthy, malaria-naïve adults 18–45 yrs	Median time to detectable parasitemia	Two hundred and sixty-three hours with ivermectin versus two hundred and sixty-two hours with placebo, all patients were cured, and adverse events included lymphocytopenia, headache, and fatigue, with one serious adverse event consisting of severe diarrhea, fever, and hypotension
Ouédraogo [22]	2013	Double-blind, placebo-controlled trial	Infected-person, clinical	NCT0160325	Balonghin, Burkina Faso	1	120	AL + IVM 1 dose (n = 40); AL + IVM 2 doses (n = 40); AL alone (n = 40)	Asymptomatic P. falciparum carriers, 15–25 yrs	Not specified	There were twenty-two adverse events, including ten mild and twelve moderate cases, consisting of abdominal pain and headache, with no serious adverse events.
Chaccour [21]	2022	Cluster RCT, 3-arm, open-label, assessor-blinded	Community MDA	NCT04966702	Mopeia, Mozambique	4	22,724	IVM 0.4 mg/kg monthly × 3 (n = 15,195); Albendazole 400 mg (n = 7,529)	15 kg, no Loa loa, not severely ill	The infection incidence rate ratio in children under five years of age	The incidence rate ratio was four point seven seven with ivermectin versus four point seven five with albendazole, adverse events included fever in six hundred and thirty-eight cases, gastrointestinal symptoms in seven hundred and five cases, vertigo in three hundred and twenty-seven cases, and itching in two hundred and ninety-nine cases, with fifteen deaths in the ivermectin group and ten deaths in the albendazole group
Some [13]	2019–2020	Phase 3, double-blind, placebo-controlled, cluster-randomised	Community MDA	NCT03967054	Diebougou, Burkina Faso	16/yr × 2 yrs	Y1: 3,532; Y2: 4,084	300 µg/kg × 3 days, 4 rounds/yr (Y1 n = 1,928; Y2 n = 2,163); Placebo (Y1 n = 1,604; Y2 n = 1,921)	90 cm height, not pregnant	weekly malaria incidence in children aged 10 years and younger, as assessed by weekly active case detection until week 16 of year 2	Repeated high-dose ivermectin mass drug administration did not reduce malaria incidence among children compared to placebo. The risk of adverse events was lower in the intervention group than in the control group
Hutchins [20]	2021–2022	Quadruple-blinded, cluster-randomised, placebo-controlled phase 3	Community MDA	NCT04844905	Bijagos, Guinea-Bissau	22	25,882	DHA-PQ + IVM 300 µg/kg/day × 3 monthly × 3 (n = 13,832); DHA-PQ + placebo (n = 12,050)	All ages (median 20 yrs)	quantitative PCR prevalence of Plasmodium falciparum parasitaemia in all age groups, during peak transmission, after the second year of intervention	Adding ivermectin to dihydroartemisinin-piperaquine mass drug administration had no additional effect on reducing malaria prevalence, also the intervention was well-tolerated
Dabira [19]	2017–2019	Open-label, cluster-randomised controlled trial	Community MDA	NCT03576313	Upper River, Gambia	—	10,638	IVM 300–400 µg/kg/day × 3 + DP (n = 4,939); Standard control (n = 5,699)	All ages	malaria prevalence by qPCR at the end of the second intervention year in November 2019, and Anopheles gambiae (s l) parous rate,	Mass drug administration of ivermectin and dihydroartemisinin-piperaquine significantly reduced malaria prevalence compared to standard control interventions, also the intervention was safe
Chaccour [18]	June-July 2008	Randomized controlled trial	Clinical/Entomology trial	—	LSHTM, UK	2	25	200 µg/kg single (n = 13); Control (n = 12)	Healthy adults	Mosquito survival post-feeding	Ivermectin reduced the lifespan of anopheles gambiae mosquitoes after day 1, but this effect disappeared after two weeks. The drug was safe

IVM = ivermectin; AL = artemether-lumefantrine; DP = dihydroartemisinin-piperaquine; DHA-PQ = dihydroartemisinin-piperaquine

Trial types: Infected-person = individual-level clinical trial; Community MDA = mass drug administration cluster trial; Entomology = mosquito mortality assay

Follow-up reflects study period for primary outcome assessment; “—” indicates not reported

Primary endpoint and efficacy & AEs summarized from abstracts and main text; outcomes reported concisely

Total N refers to all participants randomized or included in analysis; per-arm numbers are in parentheses

Safety outcomes (AEs, SAEs) include both mild/moderate and serious events as reported

### Outcome definitions and standardization

Outcome definitions were explicitly aligned with trial category. Clinical trials quantified parasitemia incidence using participant denominators over 21–28 days with individual-level adjustment. Community-level trials measured malaria incidence or prevalence using population denominators over 6–24 months with cluster adjustment where available. Entomological trials assessed mosquito mortality using mosquito denominators over 3–7 days following ivermectin exposure. Cluster-unadjusted estimates were identified as limitations. Repeated and age-specific outcome measures were retained as originally reported. Full outcome definitions are detailed in Table 2.

### Risk of bias assessment

The risk of bias was evaluated using the Cochrane RoB 2 tool [17] across five domains: randomization process, deviations from intended interventions, missing outcome data, outcome measurement, and selection of the reported result. Each study was categorized as low risk (all domains low), some concerns (at least one domain with concerns but none high risk), or high risk (at least one high-risk domain). Risk of bias due to missing results (reporting bias), as specified in the PROSPERO protocol, was assessed within the “selection of the reported result” domain of the RoB 2 tool. A summary table is provided in Supplementary Fig. 1.

### Statistical analysis

Meta-analyses were conducted only when outcomes were methodologically comparable. Studies were grouped by trial design, population level, and outcome definition before synthesis. Separate meta-analyses were performed for community-level malaria incidence, community-level prevalence, and mosquito mortality based on direct-feeding methods. Individual-level clinical trials and those reporting time-to-event parasitological outcomes were summarized narratively due to non-comparability of effect measures.

Category-specific random-effects meta-analyses were performed using the restricted maximum likelihood (REML) estimator with Hartung–Knapp adjustment in R (v 4.3.0, meta and metafor packages). Dichotomous outcomes were pooled using Mantel–Haenszel risk ratios (RRs) with 95% confidence intervals (CIs) and a 0.5 continuity correction for zero-cell values. Heterogeneity was assessed using the I2*I*2 statistic, τ2*τ*2, and the Cochran Q-test (significant at *p* < 0.10). For cluster-RCTs, analyses used reported cluster-adjusted estimates wherever available. In cases lacking intracluster correlation coefficients (ICCs), no statistical correction was possible and the limitation was acknowledged. For trials reporting repeated weekly incidence outcomes, the primary protocol-defined endpoint or the longest available follow-up was extracted to prevent unit-of-analysis errors. Studies reporting cumulative incidence (binary outcomes) were pooled separately from those presenting incidence rates derived from mass drug administration clusters.

Rate-based outcomes were synthesized using generic inverse variance methods of incidence rate ratios, while binary risk-based outcomes were pooled using random-effects RR models. Fixed-effect models were not used for inference but applied solely as diagnostic tests to identify influential studies. Small-study effects were assessed using Egger’s regression test for outcomes with ten or more contributing studies. The R code used to perform meta-analyses is provided in Supplementary File 2. full search strategies, PRISMA checklist, risk of bias tables, excluded studies list with reasons, and R code, are available in the supplementary materials.

## Results

### Study classification

Included trials [13, 18–26] were categorized into three groups from baseline by design: individual-level clinical trials (n = 3), community-based cluster/MDA trials (n = 4), and entomological trials (n = 3).

Three individual-level trials conducted in Gabon, Germany, and Burkina Faso [22, 23, 26] enrolled adults aged 18–50 years, either with Plasmodium falciparum infection or healthy, malaria-naïve volunteers. Ivermectin dosing ranged from a single 0.4 mg/kg dose to multi-day regimens of up to 0.3 mg/kg for three consecutive days.

Four large-scale mass drug administration trials were identified [13, 19–21], conducted in Mozambique, Burkina Faso, Guinea-Bissau, and The Gambia, and including large populations of children and adults. Interventions typically used ivermectin at 300–400 µg/kg, administered monthly or in multiple rounds, often co-administered with other antiparasitic agents such as DHA–PQ or albendazole. Chaccour 2025 (n = 22,724), the largest cluster-RCT, reported IRR 4.77 vs 4.75 (albendazole comparator) without events/totals and was therefore synthesized narratively, consistent with no effect direction.

Three trials focused on entomological outcomes [18, 24, 25]. The Smit trials, conducted in Kenya among adults with uncomplicated malaria, used high-dose ivermectin (300–600 µg/kg/day for three days), while Chaccour 2010 was a randomized controlled trial in the UK among healthy adults assessing mosquito survival after feeding.

### Risk of bias assessment

In the included randomized trials, the overall risk of bias was predominantly low, with only a minority of judgments rated as “some concerns” across the assessed domains. Across D1 (bias arising from the randomization process), D2 (bias due to deviations from intended interventions), D3 (bias due to missing outcome data), D4 (bias in measurement of the outcome), and D5 (bias in selection of the reported result), most studies were judged at low risk, indicating adequate sequence generation and allocation concealment, appropriate blinding or objective outcome assessment, and minimal loss to follow-up. Some studies showed concerns mainly in randomization and reporting, typically related to incomplete reporting of allocation procedures or selective reporting of outcomes, but all were judged to be at low or moderate risk of bias. Although most studies were at low risk of bias, the small number of trials and substantial heterogeneity limit the overall certainty of the evidence.

### Clinical trials

No pooled analysis for Mbassi 2023, Metzger 2020, and Ouédraogo 2014 due to heterogeneity (pre-erythrocytic prophylaxis/mosquito mortality/blood-stage clearance; CHMI/natural infection; time-to-event vs. transmission endpoints). Mbassi et al. reported numerically faster clearance (24.1 h vs. 32.0 h; HR 1.38, 95% CI 0.64–2.97); Metzger et al. found no difference in time-to-parasitemia (days 11–12); Ouédraogo et al. reported 27–35% transmission reduction via mosquito mortality.

### Community-based trials

#### Malaria prevalence

**Fig. 2:**
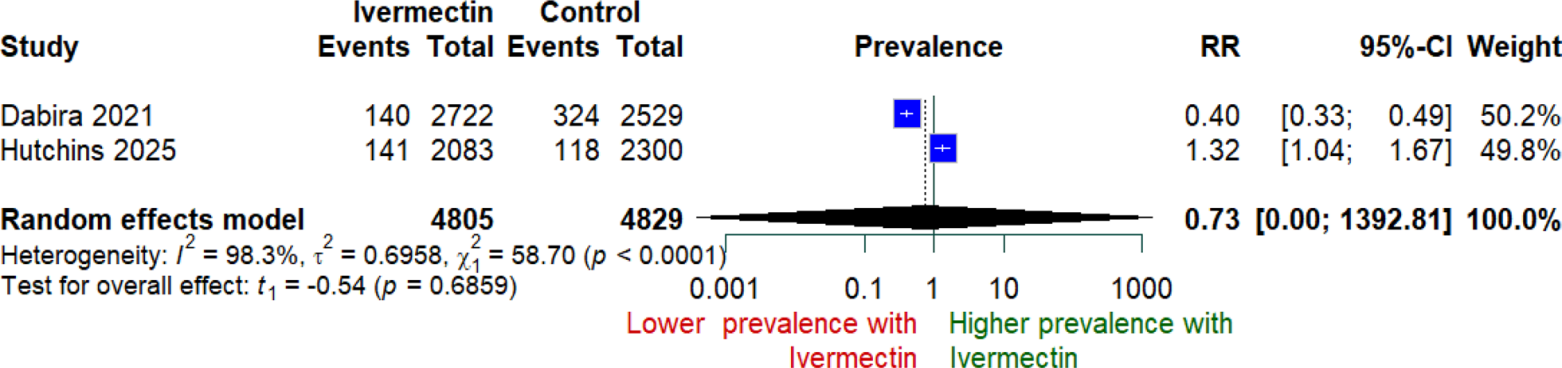
Random-effect meta-analysis for malaria prevalence

The meta-analysis included 9,634 participants from two trials (Dabira et al. and Hutchins et al.). Using a random-effects model, the pooled risk ratio (RR) for malaria prevalence was 0.73 (95% CI 0.00–1392.81; p = 0.686) **(**Fig. 2**).**, indicating no statistically significant effect. Substantial heterogeneity was present (I^2^ = 98.3%, p < 0.0001), reflecting divergent findings: Dabira et al. observed a significant reduction in prevalence (RR 0.40), whereas Hutchins et al. reported a significantly higher prevalence in the intervention group (RR 1.32). Sensitivity analysis restricted to cluster-RCTs showed consistent findings. Only one cluster-RCT (Some 2025) reported extractable events/totals for malaria incidence, yielding a risk ratio of 0.86 (95% CI 0.62–1.17). The remaining cluster-RCTs either reported incidence rate ratios without events/totals or only reported prevalence and were summarized narratively in the main analysis.

### Entomological trials

#### Mosquito mortality (direct feeding)

**Fig. 3:**
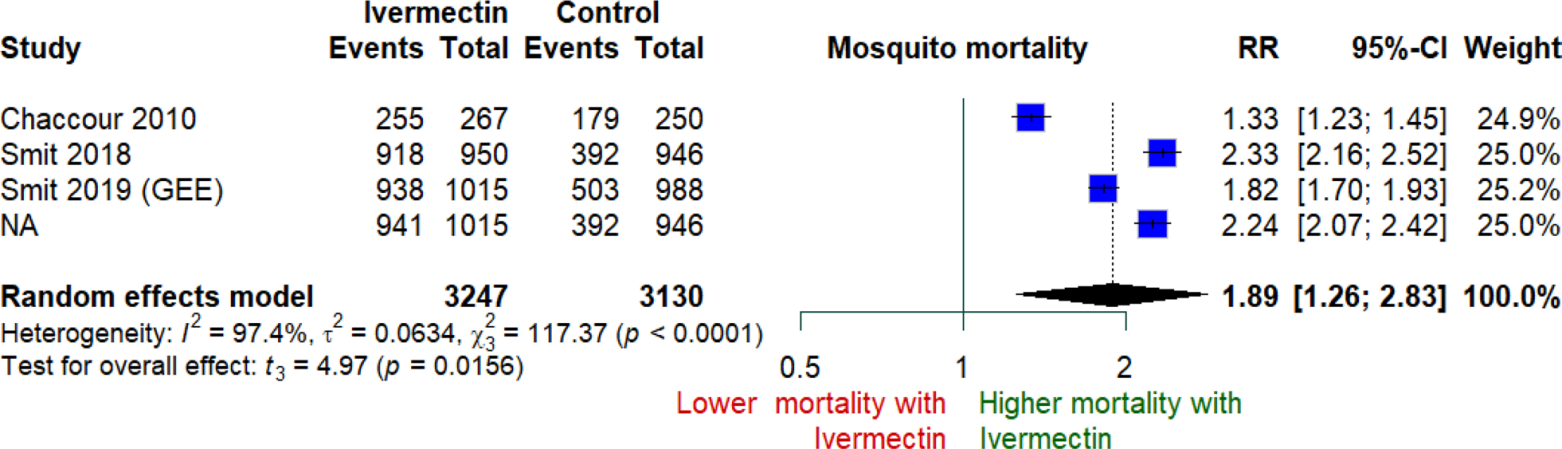
Random-effect meta-analysis for Mosquito Mortality

In entomological trials assessing mosquito mortality after direct feeding (Smit 2018, Smit 2019, Chaccour 2010), ivermectin was associated with higher mosquito mortality compared with control, with a pooled RR of 1.89 (95% CI 1.26–2.83; p = 0.02). Although heterogeneity was substantial (I^2^ = 97.4%; p < 0.001) **(**Fig. 3**),** the direction of effect was consistent across studies, indicating increased mortality in the ivermectin arm. Leave-one-out sensitivity analyses confirmed the robustness of this effect, with pooled RRs remaining statistically significant (1.89–2.11) after exclusion of any single study (supplementary Fig. 2).

### Secondary outcomes

Safety analyses included ten studies that systematically reported adverse events. No statistically significant association was observed between ivermectin administration and the incidence of any specific adverse event.

#### Systemic adverse events

Systemic outcomes, including pyrexia, fatigue, headache, arthralgia, and pharyngitis, did not exhibit a statistically significant association with ivermectin, and heterogeneity across studies was non-significant. The pooled risk ratios (RRs) and heterogeneity levels were as follows: 0.48 (95% CI [0.17; 1.38], p = 0.13) (I^2^ = 0.0%, p = 0.81) for pyrexia, 4.16 (95% CI [0.15; 118.40], p = 0.27) (I^2^ = 47.2%, p = 0.13) for fatigue, 1.27 (95% CI [0.26; 6.29], p = 0.67) (I^2^ = 22.2%, p = 0.28) for headache, 0.12 (95% CI [0.00; 3.22], p = 0.07) (I^2^ = 0.0%, p = 0.81) for arthralgia, and 1.95 (95% CI [0.05; 81.47], p = 0.26) (I^2^ = 0.0%, p = 0.79)for pharyngitis. The wide confidence intervals across analyses reflect low event rates and reduced precision. Such intervals suggest that the current evidence is insufficient to rule out rare adverse events, even though none were observed in the included studies. Overall, systemic adverse events were uncommon and showed no significant association with ivermectin (Supplementary Figs. 3–7).

#### Gastrointestinal disorders

The meta-analysis found no significant link between ivermectin use and the development of gastrointestinal events. The pooled RR was 3.01 (95% CI [0.20; 45.41], p = 0.34), with considerable heterogeneity (I^2^ = 76.1%; p < 0.001) (Supplementary Fig. 8). A leave-one-out sensitivity analysis identified Dabira et al. (2021) as a major outlier. Excluding this study reduced heterogeneity to zero and changed the pooled estimate to a directionally protective, though still non-significant, effect (RR = 0.88 (95% CI [0.44; 1.76])) (Supplementary Fig. 9)

#### Ocular and neurological events

No significant associations were found between ivermectin use and ocular or neurological outcomes. The pooled RR for visual disturbance was 7.61 (95% CI [0.58; 99.93], p = 0.08) without significant heterogeneity (I^2^ = 21.1%, p = 0.28), while that for conjunctivitis was 0.76 (95% CI [0.13; 4.48], p = 0.59) without significant heterogeneity (I^2^ = 0.0%, p = 0.8). The wide confidence intervals reflect a lack of precision and limited event reporting (Supplementary Figs. 10–11).

#### Cardiovascular and urinary disorders

Similarly, ivermectin administration showed no statistically significant association with cardiovascular or urinary outcomes. The pooled RR for cardiovascular disorders was 2.12 (95% CI [0.04; 104.56], p = 0.25) without significant heterogeneity (I^2^ = 0.0%, p = 0.78), and for urinary tract infections, 2.10 (95% CI [0.02; 210.08], p = 0.29) without significant heterogeneity (I^2^ = 0.0%, p = 0.75). The extremely wide intervals underscore the infrequency of adverse events and the resulting uncertainty in the precision of the effect estimates (Supplementary Figs. 12–13).

### Discussion

This updated systematic review and meta-analysis provides the most quantitative review of 10 RCTs that collectively included a total of 63,192 participants, assessing the impact of ivermectin on malaria. The previous Cochrane review reported limited available evidence based on only one eligible cluster-randomized controlled trial among 2712 participants, with six additional ongoing trials identified, but were not included in the quantitative synthesis as their results were unavailable at that time [14]. The current review involved newly published large-scale RCTs that studied the impact of ivermectin on the incidence and prevalence of malaria, as well as mosquito mortality. The safety profile of ivermectin was also evaluated. Despite the expanded dataset, analysis indicated no significant reduction in malaria prevalence or incidence associated with ivermectin administration when compared with standard control or placebo. Although initial studies reported a significant increase in mosquito mortality [24] and decreased mosquito survival [18], these results were not consistently reproduced in large-scale community trials and no significant reductions were observed in malaria prevalence or incidence even with repeated dosing [13, 20]. The conflict between studies could be due to variability in study scale, dosing regimens, environmental conditions, and operational implementation.

We assessed the effects of ivermectin among three distinct trial categories: individual-level clinical trials, community-based cluster/MDA trials, and entomological trials. The results varied by trial type, with individual-level clinical and community-based cluster/MDA trials showing no statistically significant reductions in malaria incidence or prevalence, suggesting insufficient evidence of clinical benefit of ivermectin. Contrary to these findings, entomological studies demonstrated a consistent increase in mosquito mortality. Differences in study design, transmission environments, ivermectin dosing regimens, follow-up durations, and outcome measures possibly accounted for the inconsistent findings and limited the comparability of results across studies.

The fixed-effect model assumes a single common effect across studies, whereas the random-effects model accounts for variability in true effects due to differences in study design, populations, and interventions. Given the substantial heterogeneity observed across included trials (I^2^ > 90%) [28], the use of a random-effects model was appropriate. Although fixed-effect models yielded statistically significant estimates in some analyses, these results are not appropriate for inference in the presence of high heterogeneity, as they may produce overly precise and potentially misleading estimates, in line with guidance from the Cochrane Handbook [15].

The pooled meta-analysis did not include the clinical trials (Mbassi 2023; Metzger 2020; Ouédraogo 2014) due to considerable variability across studies. Trials differed in study design, population characteristics, dosing regimens, co-administration with antimalarial drugs, and outcome measures. In the Mbassi study, a randomized, double-blind, placebo-controlled phase Ib/IIa design was used to evaluate ivermectin’s blood-schizonticidal efficacy in Gabonese adults with naturally acquired asymptomatic P. falciparum infection receiving doses up to 300 μg/kg over three days [26]. In contrast, Metzger et al. examined the prophylactic effect of ivermectin among healthy, malaria-naive adults in a randomized controlled human infection trial using a single dose of 0.4 mg/kg [23]. On the other hand, the study by Ouédraogo et al. was a randomized, double-blind, placebo controlled design that focused on exploring the mosquitocidal effect of ivermectin as a single or repeated dose of 200 μg/kg combined with artemether-lumefantrine to prevent malaria transmission in asymptomatic P. falciparum carriers [22]. Accordingly, this heterogeneity limited the possibility to obtain overall quantitative conclusions.

The community-based trials (Some 2025; Chaccour 2025) measured the effect of ivermectin mass drug administration on malaria incidence. However, the heterogeneity between studies precluded a pooled meta-analysis. Trials exhibited variations in study design, setting, age groups, and treatment coverage rates. The RIMDAMAL II by Some et al. was a phase 3, double-blind, placebo-controlled, parallel-group trial in Burkina Faso where ivermectin was administered as a monthly oral high-dose MDA (three daily doses of approximately 300 μg/kg) over two consecutive rainy seasons [13]. The population coverage of at least one dose ranged from 68 to 74% and the incidence of malaria was measured in a cohort of children aged ≤ 10 years [13]. In contrast, the BOHEMIA trial by Chaccour et al., carried out in Mozambique, aimed to evaluate the incidence of malaria across a cohort of children under five years receiving a single 400 mcg/kg dose of ivermectin, once a month for three months [21]. The trial aimed to achieve 80% treatment coverage, but it was disrupted by severe floods that led to low coverage and limited the clear interpretation of the efficacy data [21]. In addition, trials varied in the timing of drug administration, with the RIMDAMAL II trial implementing monthly doses of ivermectin during the rainy seasons (July–October) [13], while the BOHEMIA trial experienced delayed implementation with respect to the transmission period [21].

The pooled analysis from studies examining the effect of ivermectin on malaria prevalence (Dabira 2021; Hutchins 2025) showed non-statistically significant effects. Dabira et al. observed a significant reduction in prevalence with mass drug administration of ivermectin and DHA–PQ [19], while Hutchins et al. found a higher prevalence in the ivermectin treatment group [20]. This can be explained by important differences in study design, setting, population characteristics, and transmission intensity. The open-label Gambian study by Dabira et al. utilized repeated rounds of mass drug administration with ivermectin and DHA–PQ in a setting with high coverage of standard control interventions [19]. Compared with this, Hutchins et al. assessed the addition of ivermectin to DHA–PQ MDA in the MATAMAL study, which was a quadruple-blinded, placebo-controlled trial performed in Guinea-Bissau, a setting with extensive population mixing [20]. Besides, there were notable differences in treatment coverage between studies. The Gambian trial attained coverage rates of 51.4–85.5% for DHA–PQ and 40.0–67.6% for ivermectin [19], relative to a coverage rate between 54.5 and 75.4% in the ivermectin plus DHA–PQ treatment arm for the MATAMAL trial [20]. These variations could have influenced the results and limited the detectability of a reliable pooled effect.

In entomological trials (Smit 2018; Smit 2019; Chaccour 2010), our findings showed substantial heterogeneity between studies. Despite this, results reported a consistent increase in mosquito mortality following ivermectin administration. Sensitivity analyses confirmed the robustness of the results, supporting the adjunctive role of ivermectin in vector control strategy. Nevertheless, this should be interpreted with caution, since the variation between studies makes the single-effect assumption unreliable. The heterogeneity is evident across ivermectin dosing regimens, mosquito feeding methods, and timing of mosquito exposure after drug administration. For example, Chaccour et al. investigated the effect of a single oral dose of 200 μg/kg of ivermectin in healthy volunteers and assessed mosquito mortality after the feeding process [18]. Smit et al. (2018) examined the mosquitocidal efficacy of 3-day courses of high-dose ivermectin (300 and 600 μg/kg per day) in conjunction with DHA–PQ in Kenyan adults with uncomplicated malaria [24]. The nested sub-study (Smit et al., 2019) used similar dosing but addressed methodological differences in mosquito feeding [25]. Also, the included trials showed variability in mosquito feeding techniques. In the Chaccour trial, mosquitoes were applied directly to the forearm of each volunteer for 10–12 min on the subsequent day after ivermectin administration, and mosquito mortality was recorded daily for 12 days [18]. Alternatively, Smit et al. (2018) employed membrane feeding and assessed mosquito survival daily for up to 28 days after feeding [24]. Smit et al. (2019), in contrast, applied both direct skin and membrane feeding assays on post-treatment day 7, and examined mosquito survival daily for 28 days post-feeding [25].

As for the safety of ivermectin, our meta-analysis found that ivermectin was generally safe and well-tolerated, with most adverse events being mild to moderate in severity and not related to ivermectin administration. This suggests that ivermectin can be administered safely; however, more evidence is still needed to establish its efficacy. Differences in concomitant antimalarial medications may act as important effect modifiers [22, 24]. Co-administered therapies such as dihydroartemisinin–piperaquine or artemether–lumefantrine can independently reduce parasitemia and transmission, potentially masking or enhancing the observed effect of ivermectin [22, 24]. This interaction represents an important source of heterogeneity and should be carefully considered in the interpretation of results [19, 20].

An important aspect in interpreting the current evidence is the need to distinguish between ivermectin’s individual-level effects and its impact among populations. While ivermectin demonstrates clear mosquitocidal effects at the individual level, translating these effects into measurable reductions in malaria transmission at the population level remains challenging [13, 20, 21]. This is influenced by factors such as treatment coverage, timing relative to transmission peaks, ecological variability, and concurrent malaria control interventions, all of which may attenuate observable epidemiological impact [19, 24]. Six studies explored the effects of ivermectin at the individual level and determined outcomes such as mosquito mortality, including measurement of mosquito survival after feeding on treated participants [18, 22–26]. By contrast, four other clinical trials evaluated the impact of ivermectin at the population level [13, 19–21], during which the drug was administered to entire communities using mass drug administration to assess outcomes like malaria prevalence or incidence. Evidence showed a clear mosquitocidal effect of ivermectin at the individual level, with trials reporting increased mosquito mortality. However, community-based trials revealed mixed results on ivermectin’s effect on malaria transmission at the population level. For instance, the MATAMAL and RIMDAMAL II trials reported limited reductions in the prevalence or incidence of malaria despite measurable effects on mosquito survival (Some 2025; Hutchins 2025). Hence, these results illustrate the difficulty of translating individual-level mosquitocidal effects into measurable reductions in malaria transmission across populations. The epidemiological impact of ivermectin depends on multiple factors such as treatment coverage, the timing of drug administration, and concurrent malaria control strategies.

The prior Cochrane review included both children and adults but restricted inclusion to cluster-randomized trials, ultimately identifying only one study eligible for quantitative synthesis [14]. In contrast, our review included both cluster and individual randomized controlled trials, thereby expanding the available evidence base. These differences in inclusion criteria should be considered when comparing findings between the two reviews [14]. Only one study met the inclusion criteria and was included in the main analysis, the RIMDAMAL trial conducted in Burkina Faso [27], and results showed no effect of ivermectin on the cumulative incidence of malaria in the cohort of children (RR 0.86, 95%CI 0.62–1.17) [14]. The evidence was of very low certainty as the effect estimate was based on a single trial with a high risk of bias [14].

In contrast, our review covered additional randomized controlled trials, including non-cluster RCTs, thus expanding the scope of available evidence and providing a more comprehensive assessment of ivermectin’s effects. The cluster-randomized trial by Foy et al. (RIMDAMAL, Burkina Faso) [27], which was included in the Cochrane review [14], was not included in the quantitative meta-analysis of the present review because the primary malaria outcomes were measured only in children aged ≤ 5 years [27], whereas our review focused on adult clinical outcomes. Most children in the study were not directly treated due to height-based eligibility restrictions, and the observed reduction in malaria incidence resulted primarily from indirect effects on mosquito populations [27]. Additionally, outcomes were reported at the cluster level, preventing extraction of individual-level data required for pooling [27]. Despite this, the trial is summarized narratively to provide context regarding repeated mass ivermectin administration and its potential effects on malaria transmission.

### Strengths and limitations

The strengths of our study are as follows: (1) We included large, community-based RCTs carried out among a range of endemic settings, (2) It is the first systematic review and meta-analysis focusing on adult population, providing the most up to date synthesis of available data, (3) We performed sensitivity analysis to assess the robustness of our findings. However, our review had several limitations. Firstly, the number of trials per study design was relatively small. Secondly, there was substantial heterogeneity across studies. Beyond this, no studies provided cost-effectiveness data, which is an essential consideration for informing policy decisions. Moreover, some of the trials were available only as preprints, reducing confidence in the results. Finally, the short follow-up periods in most studies could have underestimated long-term outcomes.

### Clinical implications

The results of our meta-analysis revealed that ivermectin had no significant impact on malaria outcomes, however, the findings have important practical implications. Current evidence does not support the efficacy of administering ivermectin for malaria control. Nonetheless, ivermectin may still serve as an adjunct intervention with established malaria control strategies such as insecticide-treated nets, indoor residual spraying, vaccines, and seasonal malaria chemo prevention [29, 30]. In addition, adjusting the dosing regimen [31] and timing according to peak transmission intervals might increase the efficacy of ivermectin [12, 13], though additional high-quality research is necessary. Furthermore, the effectiveness of ivermectin may vary according to the local malaria transmission intensity [32], population adherence [33], and the composition of mosquito species [34, 35], highlighting the importance of implementing approaches based on the local setting. As a result, policymakers and practitioners should cautiously interpret the existing evidence and take these factors into consideration when developing malaria control programs.

### Recommendations for future research

The included trials in our review demonstrated substantial heterogeneity in ivermectin dosing and methodological techniques [13, 18–26], limiting the comparability between studies and making it difficult to establish optimal dosing strategies. Substantial variability in dosing regimens, treatment schedules, and study methodologies was observed across included trials. This variability reflects the absence of standardized dosing strategies, highlighting the need for pharmacokinetic–pharmacodynamic optimization and standardized protocols in future studies. Therefore, we recommend developing standardized protocols Therefore, we recommend developing standardized protocols for future research with the aim to optimize ivermectin dosing and integrate pharmacokinetic-pharmacodynamic modeling to provide effective strategies for sustained mosquito control [11]. Moreover, current studies are limited by short follow-up periods, thus, research should also focus on conducting larger cluster-RCTs with follow-up durations of at least 12 months to assess the long-term effects of ivermectin. Treatment effectiveness and safety profiles may also differ across age groups, for this reason, stratified analyses for the pediatric population are essential, as children are highly susceptible to malaria [36]. Finally, examining the optimal timing for seasonal mass drug administration of ivermectin relative to malaria transmission is crucial, given that the effect of vector control and drug-based interventions is determined by their timing in relation to peak transmission [37].

### Policy implications

Current evidence does not support ivermectin as a standalone intervention for malaria control, as large-scale community trials have not shown consistent reductions in malaria incidence or prevalence despite its demonstrated mosquitocidal activity in laboratory settings. Therefore, ivermectin should not replace proven strategies such as insecticide-treated bed nets, indoor residual spraying, seasonal chemoprevention, vaccination programs, or effective case management. Nevertheless, it may hold potential as a complementary tool within integrated vector management [29, 30], particularly if advances in pharmacokinetics—such as long-acting formulations or sustained-release systems—can maintain mosquitocidal concentrations safely over longer periods [38, 39]. Any policy consideration should be informed by robust pharmacokinetic-pharmacodynamic modeling, operational feasibility studies, ethical evaluation of mass drug administration, community adherence assessments, resistance risk monitoring, and coordination with existing malaria elimination initiatives. For now, ivermectin-based interventions should remain within the research sphere until stronger, high-quality evidence confirms consistent, population-level benefits.

### Conclusion

Our analysis illustrates that ivermectin, based on current evidence, does not have a statistically significant effect on malaria outcomes. These findings emphasize the necessity of further trials to evaluate its potential role within malaria control initiatives.

## Supplementary Information

Additional file1

Additional file2

## Data Availability

The datasets used and/or analyzed during the current study are available from the corresponding author on reasonable request.
