# Supplementary material for: Efficacy of ivermectin for malaria vector control: a systematic review and meta-analysis of randomized clinical trials
Source: Malar J. 2026 Mar 31;25:217. doi: 10.1186/s12936-026-05895-z (PMC13188585; doi:10.1186/s12936-026-05895-z)
Supplement: Supplementary file 2 — Additional file2 [file 12936_2026_5895_MOESM2_ESM.docx]

**Supplementary Appendix**

|  | **Page** |
| --- | --- |
| **Supplementary Table 1.** Search strategy for each database. | 2 |
| **Supplementary Figure 1.** Risk of bias assessment using ROB-2 tool. | 3 |
| **Supplementary Figure 2.** Leave-one-out analysis for Mosquito Mortality | 6 |
| **Supplementary Figure 3.** Random-effect meta-analysis of Pyrexia rates | 7 |
| **Supplementary Figure 4.** Random-effect meta-analysis of Fatigue rates | 8 |
| **Supplementary Figure 5.** Random-effect meta-analysis of Headache rates | 9 |
| **Supplementary Figure 6.** Random-effect meta-analysis of Arthralgia rates | 10 |
| **Supplementary Figure 7.** Random-effect meta-analysis of Pharyngitis rates | 11 |
| **Supplementary Figure 8.** Random-effect meta-analysis of Gastrointestinal Disorders rates | 12 |
| **Supplementary Figure 9.** Leave-one-out analysis for Gastrointestinal Disorders rates | 13 |
| **Supplementary Figure 10.** Random-effect meta-analysis of Visual disturbance | 14 |
| **Supplementary Figure 11.** Random-effect meta-analysis of Conjunctivitis | 15 |
| **Supplementary Figure 12.** Random-effect meta-analysis of cardiovascular disorder rates | 16 |
| **Supplementary Figure 13.** Random-effect meta-analysis of Urinary tract infection rates | 17 |
| **Supplementary Table 2.** Table of excluded studies | 18 |
| **Supplementary Table 3.** Data table | 19 |

**Supplementary Table 1. Search strategy for each database.**

| **Database** | **Search query** | **Filter** | **Search results** |
| --- | --- | --- | --- |
| PubMed | (("Malaria"[MeSH Terms] OR malaria[Title/Abstract] OR plasmodium[Title/Abstract] OR anopheles[Title/Abstract] OR mosquito*[Title/Abstract])  AND  ("Ivermectin"[MeSH Terms] OR ivermectin[Title/Abstract] OR abamectin[Title/Abstract] OR avermectin[Title/Abstract]))  AND  ("Albendazole"[MeSH Terms] OR albendazole[Title/Abstract] OR placebo[Title/Abstract] OR "standard treatment"[Title/Abstract])  AND  (randomized controlled trial[Publication Type] OR randomi* [Title/Abstract] OR placebo[Title/Abstract] OR trial[Title/Abstract])  AND  ("malaria incidence"[Title/Abstract] OR "malaria transmission"[Title/Abstract] OR "adverse effects"[Title/Abstract] OR "adverse events"[Title/Abstract] OR outcome*[Title/Abstract])) | All fields | 16 |
| Scopus | (TITLE-ABS-KEY(malaria OR plasmodium OR anopheles OR mosquito*))  AND  (TITLE-ABS-KEY(ivermectin OR abamectin OR avermectin))  AND  (TITLE-ABS-KEY(albendazole OR placebo))  AND  (TITLE-ABS-KEY("randomized controlled trial" OR randomized OR placebo OR randomly))  AND  (TITLE-ABS-KEY("malaria incidence" OR "adverse effects" OR "adverse events" OR outcome*)) | Article  title, Abstract, Keywords | 32 |
| Web of Science | TS=(malaria OR plasmodium OR anopheles OR mosquito*)  AND  TS=(ivermectin OR abamectin OR avermectin)  AND  TS=(albendazole OR placebo)  AND  TS=("randomized controlled trial" OR randomized OR placebo OR randomly)  AND  TS=("malaria incidence" OR "adverse effects" OR "adverse events" OR outcome*) | Topic | 28 |
| Cochrane | ([mh "Malaria"] OR malaria OR plasmodium OR anopheles OR mosquito*)  AND  ([mh "Ivermectin"] OR ivermectin OR abamectin OR avermectin)  AND  ([mh "Albendazole"] OR albendazole OR placebo)  AND  (randomized controlled trial OR randomized OR placebo OR randomly)  AND  ("malaria incidence" OR "adverse effects" OR "adverse events" OR outcome*) | Title, Abstract, Keyword | 31 |
| Total | 107 |  |  |
| After Duplicate Removing (By EndNote xx) | 97 |  |  |

**Supplementary Figure 1.** Risk of bias assessment using ROB-2 tool.


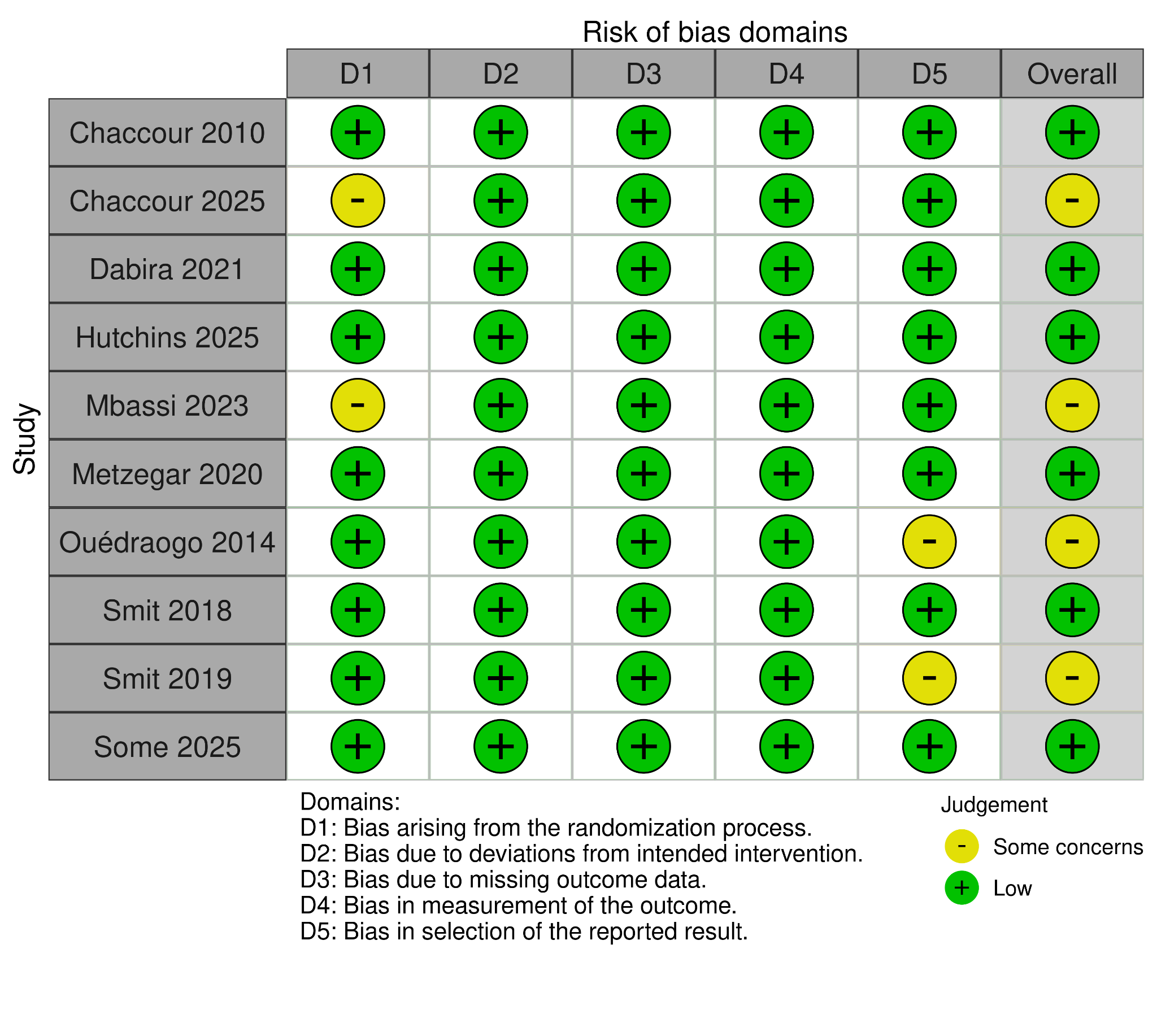


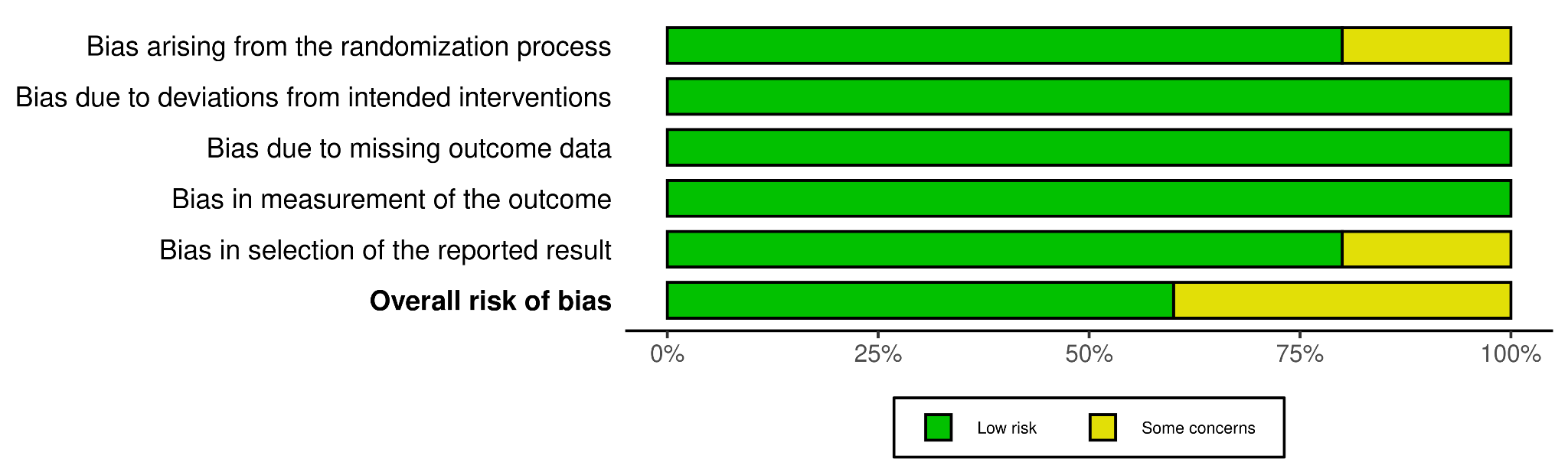


**Supplementary Figure 2.** Leave-one-out analysis for mosquito Mortality
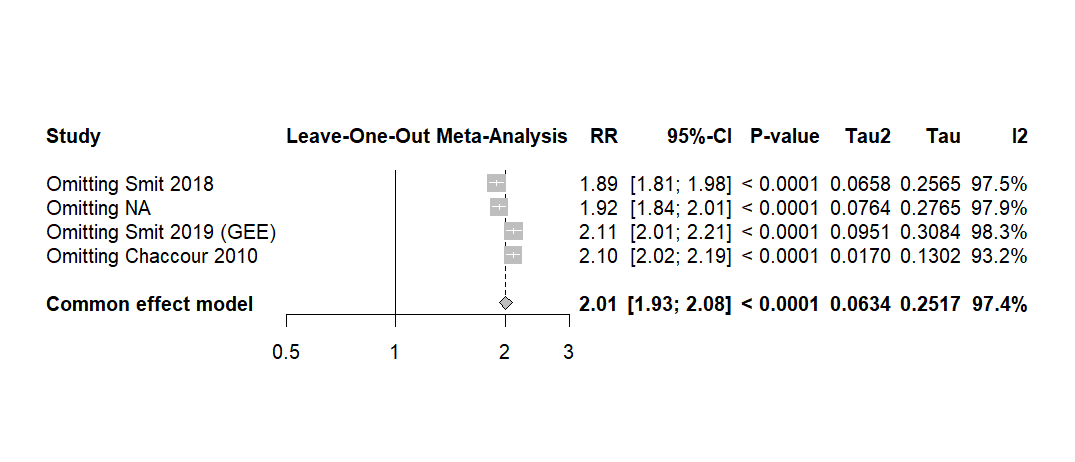


**Supplementary Figure 3.** Random-effect meta-analysis of Pyrexia rates


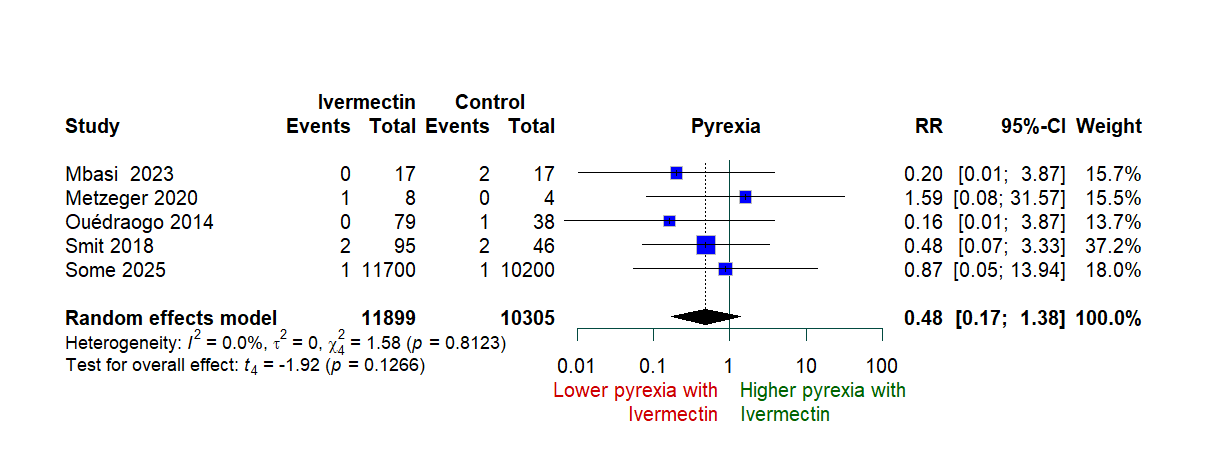


**Supplementary Figure 4.** Random-effect meta-analysis of Fatigue rates
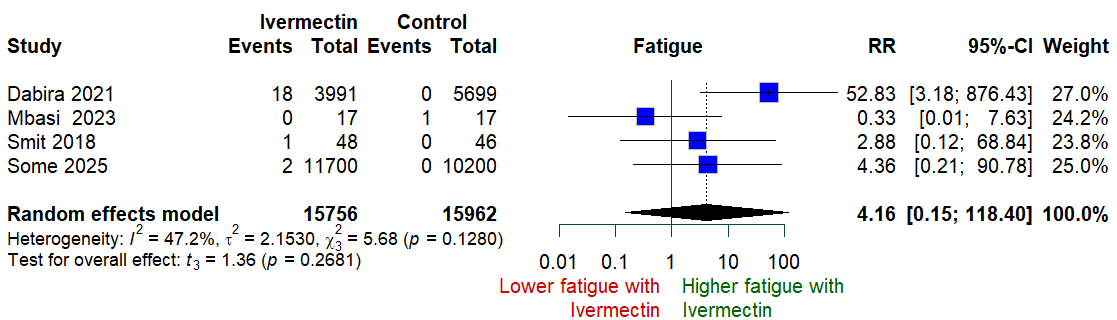


**Supplementary Figure 5.** Random-effect meta-analysis of Headache rates


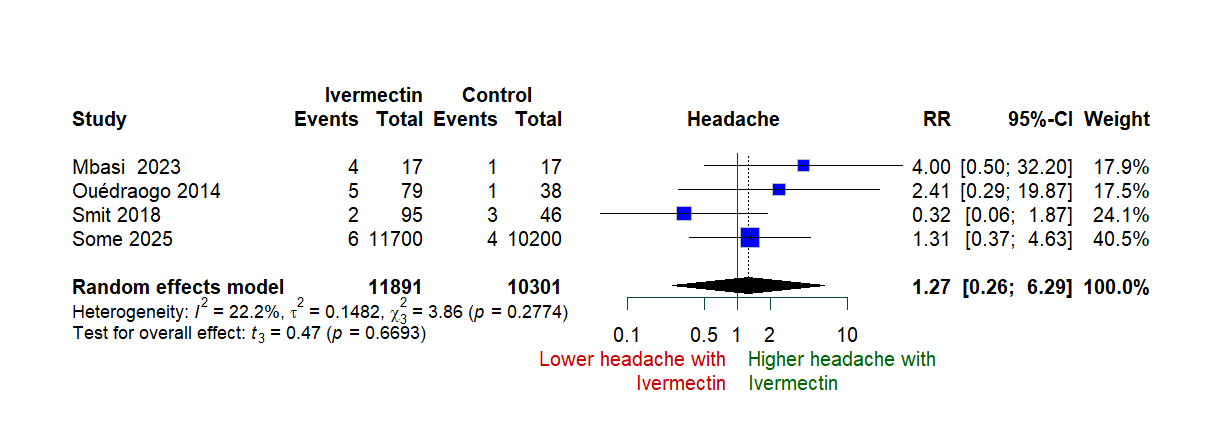


**Supplementary Figure 6.** Random-effect meta-analysis of Arthralgia rates


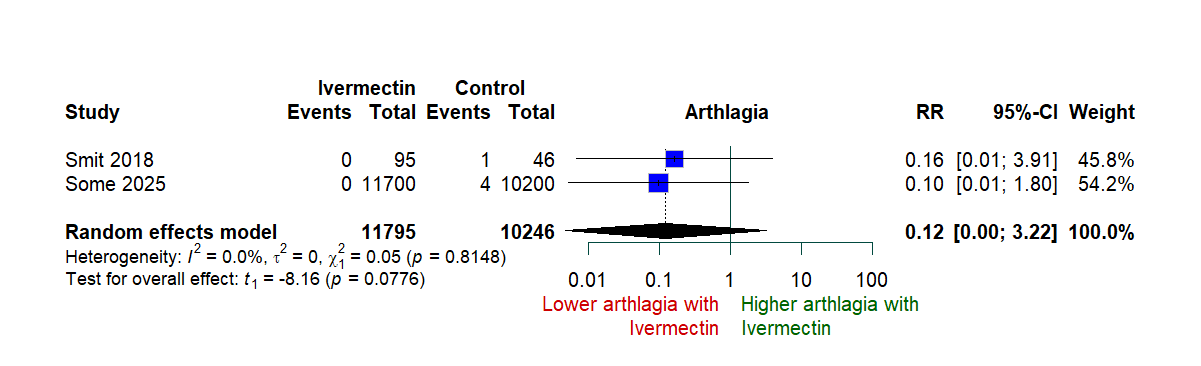


**Supplementary Figure 7.** Random-effect meta-analysis of Pharyngitis rates


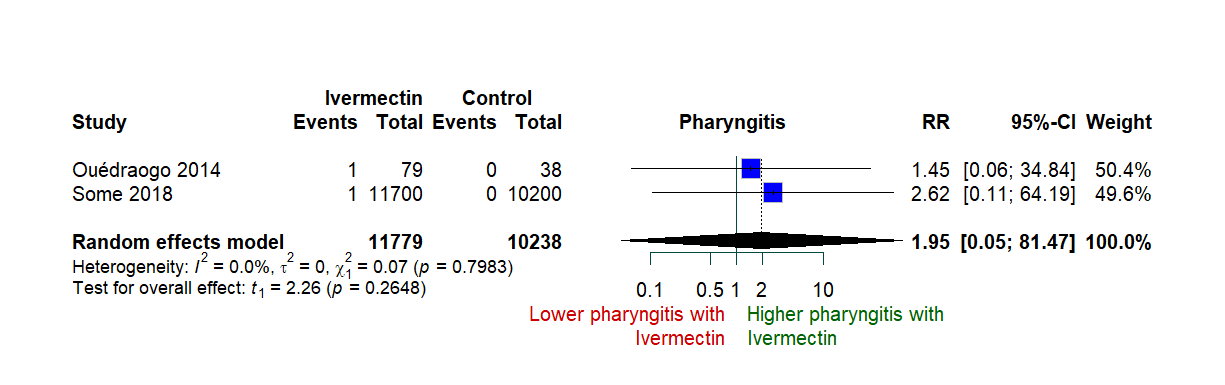


**Supplementary Figure 8.** Random-effect meta-analysis of Gastrointestinal Disorders rates


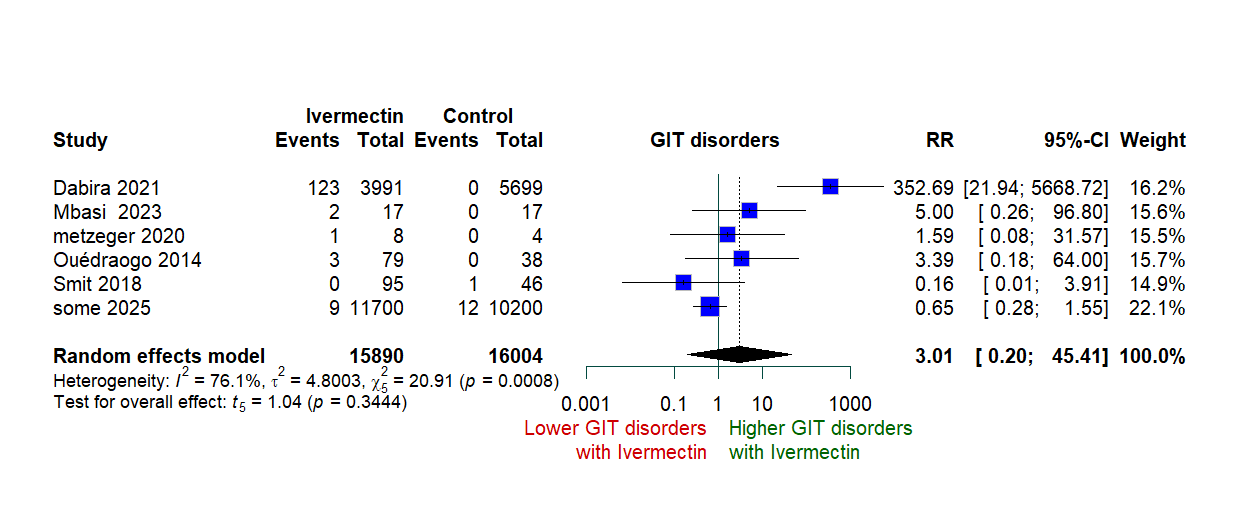


**Supplementary Figure 9.** Leave-one-out analysis for Gastrointestinal Disorders rates


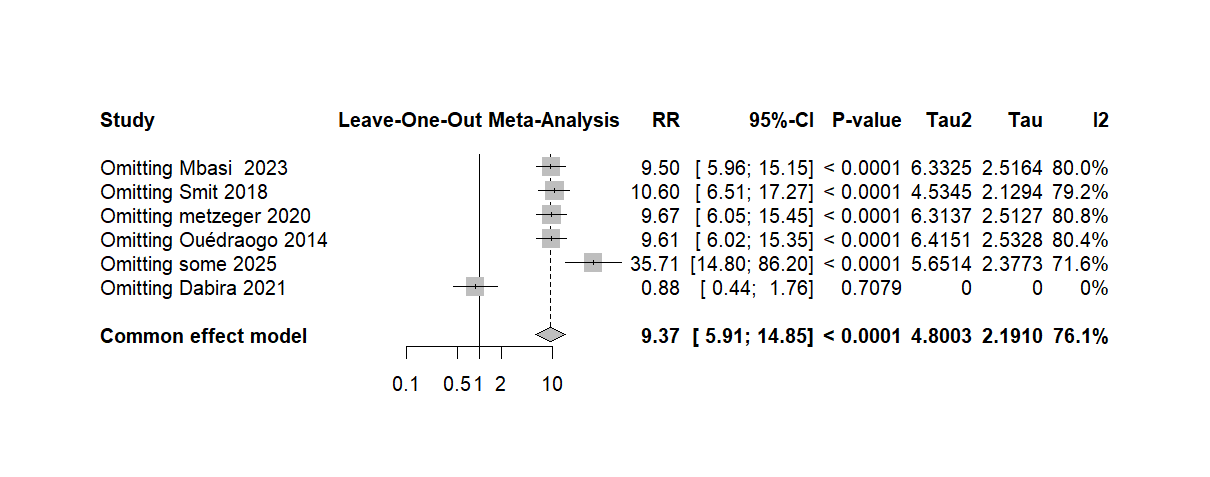


**Supplementary Figure 10.** Random-effect meta-analysis of Visual disturbance


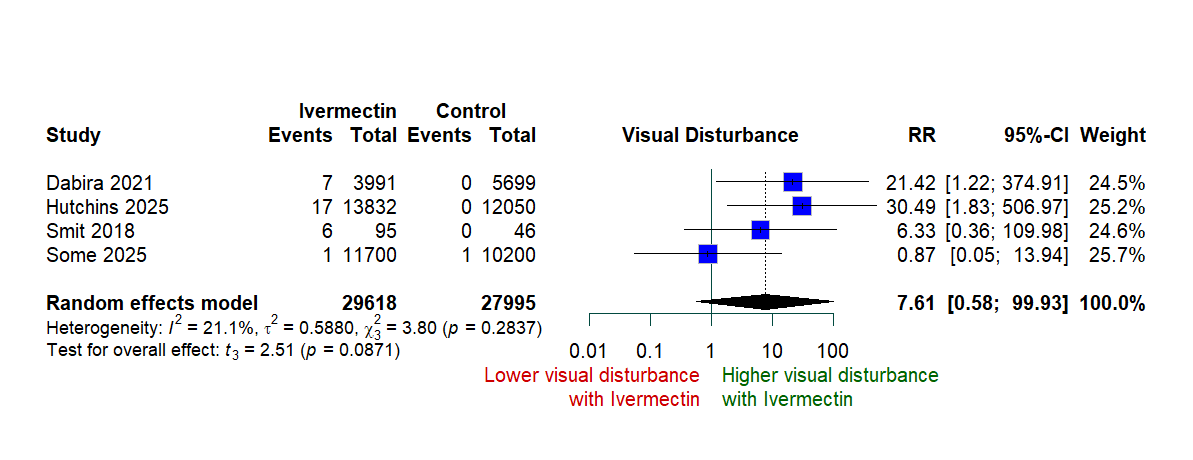


**Supplementary Figure 11.** Random-effect meta-analysis of Conjunctivitis


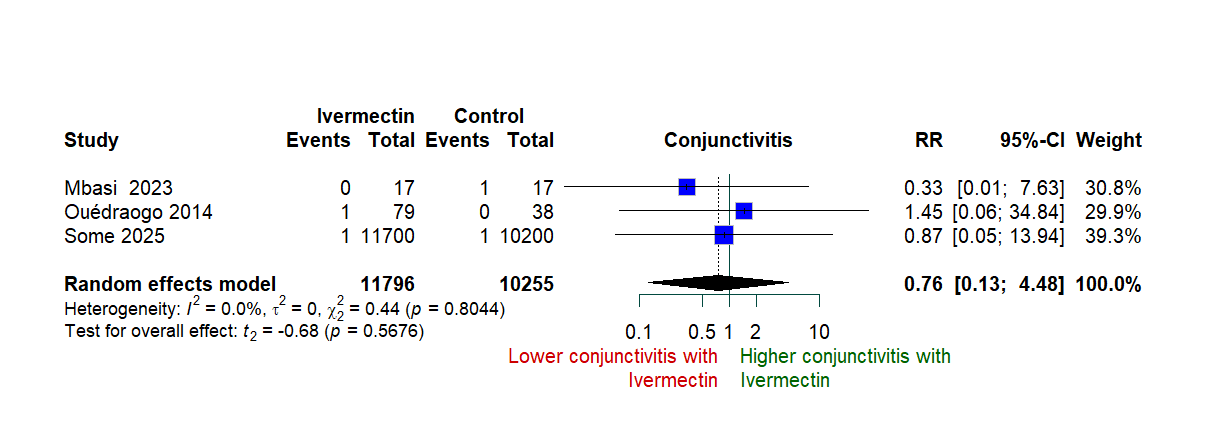


**Supplementary Figure 12.** Random-effect meta-analysis of Cardiovascular Disorders rates


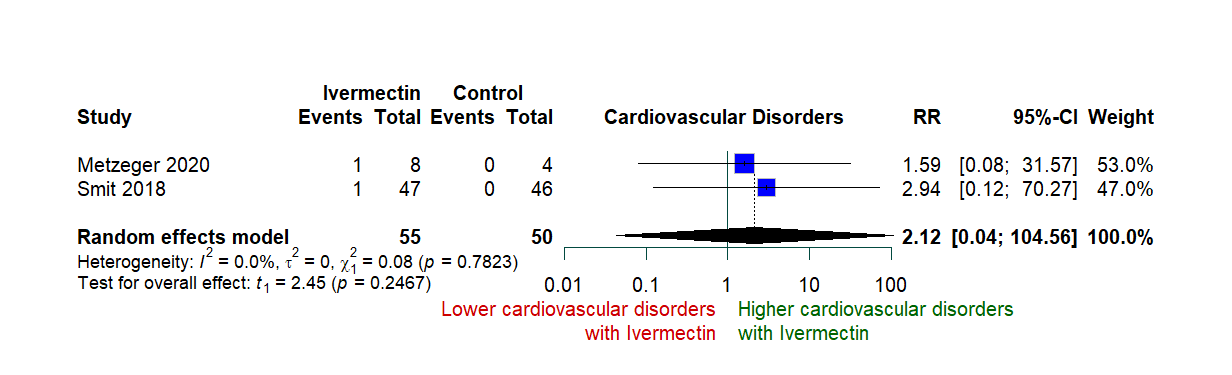


**Supplementary Figure 13.** Random-effect meta-analysis of Urinary tract infection rates


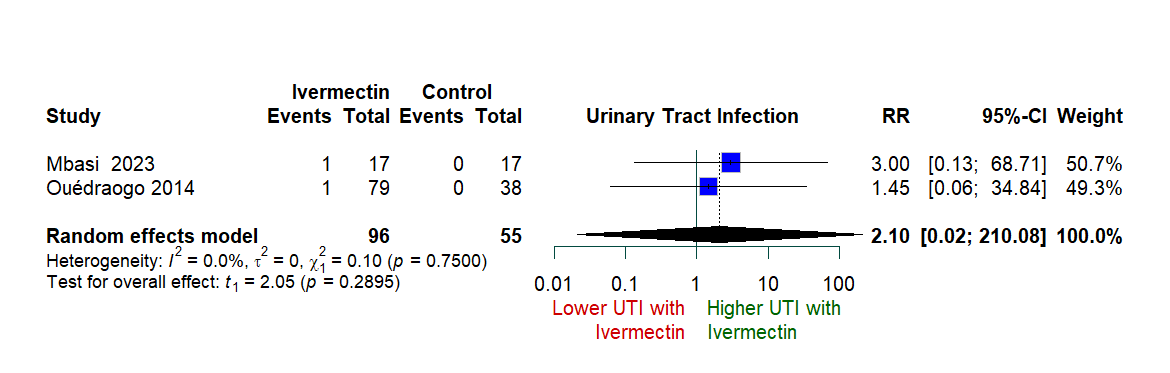


**Supplementary Table 2.** Table of excluded studies

| **Study ID** | **Reason of exclusion** |
| --- | --- |
| Alout 2014 | Wrong design, observational study |
| Chaccour 2023 | Protocol |
| Derua 2015 | Wrong population, healthy volunteers |
| Mekuriaw 2019 | Wrong population, healthy volunteers |
| Pinilla 2018 | Wrong population, healthy volunteers |
| Foy 2019 | Primary outcomes reported only in children < 5 years; adult outcomes not available for pooling; cluster-level data prevented extraction for meta-analysis |
| Fabrice 2025 | Wrong population, healthy participants |
| Foy 2023 | Protocol |
| Foley 2000 | Wrong population, healthy volunteers |

**Supplementary Table 3.** Data Table

| **Outcome** | **Study** | **Intervention** | **Event** | **Total** | **Control Event** | **Control Total** |
| --- | --- | --- | --- | --- | --- | --- |
| **Mosquito Mortality: Direct Feeding** |  |  |  |  |  |  |
|  | Smit 2018 | IVM 600 μg/kg/day ×3 | 918 | 950 | 392 | 946 |
|  | Smit 2019 (GEE) | IVM 300 μg/kg/day ×3 | 941 | 1015 | 392 | 946 |
|  | Chaccour 2010 | IVM 200 μg/kg | 255 | 267 | 179 | 250 |
| **Malaria Prevalence** |  |  |  |  |  |  |
|  | Hutchins 2025 | IVM 300 μg/kg/day ×3 | 141 | 2083 | 118 | 2300 |
|  | Dabira 2021 | IVM 300–400 μg/kg | 140 | 2722 | 324 | 2529 |

†Smit 2018: High-dose regimen (600 μg/kg/day ×3 days) with DHA-PQ; membrane feeding assay

‡Smit 2019: GEE-adjusted analysis; direct skin + membrane feeding on post-treatment day 7

§Chaccour 2010: Single 200 μg/kg dose; direct skin feeding 24h post-dose
